# Citizen Science and Monitoring Forest Pests: a Beneficial Alliance?

**DOI:** 10.1007/s40725-022-00176-9

**Published:** 2022-11-25

**Authors:** Maarten de Groot, Michael J. O. Pocock, Jochem Bonte, Pilar Fernandez-Conradi, Elena Valdés-Correcher

**Affiliations:** 1 Slovenian Forestry Institute, Večna Pot 2, 1000 Ljubljana, Slovenia; 2 UK Centre for Ecology & Hydrology, Wallingford, Oxfordshire OX10 8BB UK; 3 Flanders Research Institute for Agriculture, Fisheries and Food (ILVO), Burg. Van Gansberghelaan 96, 9820 Merelbeke, Belgium; 4 INRAE, UR629 Recherches Forestières Méditerranéennes (URFM), 84914 Avignon, France; 5 BIOGECO, INRAE, University Bordeaux, Cestas, Bordeaux, France

**Keywords:** Forest health, Community science, Forest management, Awareness raising, Forest protection

## Abstract

**Purpose of the Review:**

One of the major threats to tree health, and hence the resilience of forests and their provision of ecosystem services, is new and emerging pests. Therefore, forest health monitoring is of major importance to detect invasive, emerging and native pest outbreaks. This is usually done by foresters and forest health experts, but can also be complemented by citizen scientists. Here, we review the use of citizen science for detection and monitoring, as well as for hypothesis-driven research and evaluation of control measures as part of forest pest surveillance and research. We then examine its limitations and opportunities and make recommendations on the use of citizen science for forest pest monitoring.

**Recent Findings:**

The main opportunities of citizen scientists for forest health are early warning, early detection of new pests, monitoring of impact of outbreaks and scientific research. Each domain has its own limitations, opportunities and recommendations to follow, as well as their own public engagement strategies. The development of new technologies provides many opportunities to involve citizen scientists in forest pest monitoring. To enhance the benefits of citizen scientists’ inclusion in monitoring, it is important that they are involved in the cocreation of activities.

**Summary:**

Future monitoring and research may benefit from tailor-made citizen science projects to facilitate successful monitoring by citizen scientists and expand their practice to countries where the forest health sector is less developed. In this sense, citizen scientists can help understand and detect outbreaks of new pests and avoid problems in the future.

## Introduction

Forests are complex ecosystems that provide many ecosystem services, but are under pressure for several reasons like climate change or emerging and invasive alien pests [1–4]. Climate change has increased the risks to forest health, as trees weaken as a result of drought, windthrow, or other large-scale climatic events [5]. Impacts from pests and diseases are also increasing: there new arrivals of potential invasive alien pest species [6], and increasing outbreaks of native species (‘emerging and irruptive pests’). Combined with these threats, humans are increasingly cutting forests unsustainably or accommodating potential invasive pests and irruptive species into forests [7]. It is important to increase monitoring, protective measures and actions related to forest health, including the detection of pest outbreaks and invasive pests, in order to avoid ecological and economic damage [8].

**Fig. 1:**
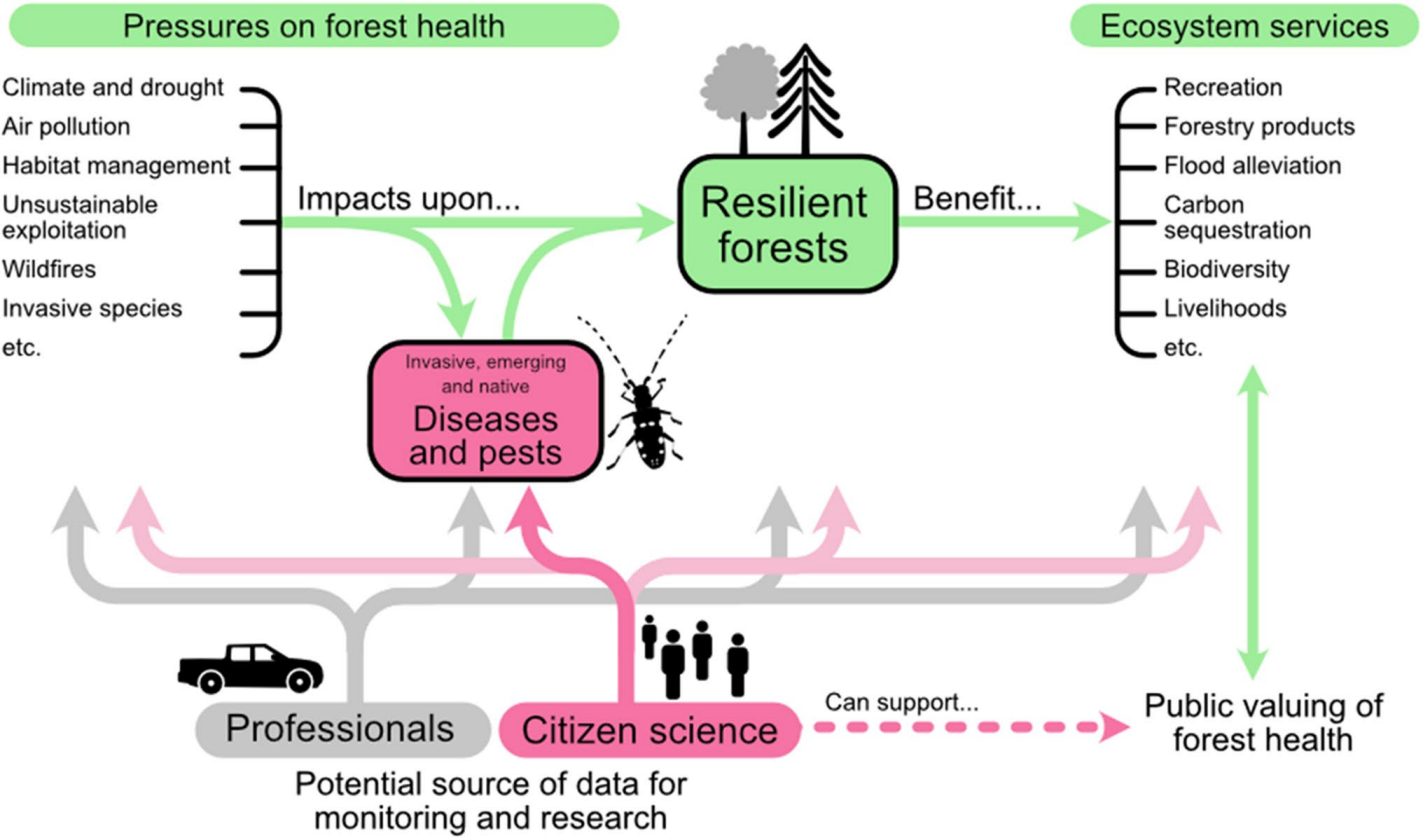
Overview of how citizen science can help with the monitoring of invasive, emerging and native pest and diseases to maintain forest health and the provision of ecosystem services through its monitoring. The focus of this paper is the role of citizen science on forest pests and diseases (shown in dark pink) and how it fits into the bigger perspective of resilient forests

Forest health can be defined as an integration of utilitarian and ecosystem measures of forest conditions and functions, applied to a range of spatial scales [9]. This means that even a healthy forest is expected to have tree mortality due to biotic and abiotic factors (i.e. including ‘pests’ and diseases) and to exhibit resilience around a dynamic equilibrium of the forest structure and function [9, 10]. When its status exceeds this natural expectation, this can be regarded as an ‘unhealthy’ forest. There are many drivers of forest health, including forest pests and diseases (Fig. 1; [11]). Pests and diseases can be a response of other drivers, e.g. climate change, and so can be an indicator of the impacts of that driver, or they can be a direct driver of change in the forests themselves [12].

In the current paper, we focus on forest insect pests and diseases. These can be among the most destructive causes of changes in tree and forest health. For instance, the European spruce bark beetle in Central Europe [13] or the Emerald ash borer in North America and Russia [14] have each killed millions of trees. Forest pests include invasive alien species (i.e. new introductions to a region) and emerging and native irruptive pests [8]. In order to address the issue of forest pests, an integrated approach of forest pest management is needed, requiring early detection, monitoring populations and assessing the impact of interventions, all informed by species research. This approach primarily entails eradication, i.e. early warning and early detection of population changes, followed by containment and management of outbreaks [8]. To achieve this, monitoring data from forests is required to support timely action, where this is possible, and to gain a better understanding of forest health.

Monitoring of and research on forest pests is typically done by foresters or forest health experts [15]. However, citizen science is a valuable tool for monitoring and research; it has demonstrated potential to support monitoring of and research on forest pests, and it has the additional benefit of engaging the public in forest health [16]. 

Citizen science is defined as the voluntary involvement of people in scientific research and monitoring [17]; their involvement usually includes collection of data, but can also include question formulation at project inception, co-design of methodologies, data analysis and disseminating results. Citizen science is diverse, covering approaches from structured surveys to opportunistic recording, approaches with specialist volunteer audiences to mass participation and simple to elaborate approaches [18]. Despite the diversity of approaches, they all share the outcome of providing data that is valuable for scientific research and monitoring. This means that citizen science requires a clear purpose and the use of appropriate tools and methodologies [19]. Citizen science should be evaluated against its ability to provide data that are fit for their intended purpose [20].

Citizen science can engage a wide range of different people, so should not be restricted to recruiting the ‘general public’. For instance, citizen science can involve the voluntary participation of any public audience, including small woodland owners and recreational users of forests. Experts and researchers may, of course, also be included in the monitoring as volunteers. Understanding the motivations of those involved is important. Recording wildlife through citizen science has a long history, especially in northwestern Europe (e.g. [21]). Often, citizen science is regarded as an intentional leisure activity, and recent studies have suggested that it particularly appeals to those who are relatively affluent and well-educated [22, 23]. This can be valuable when the focus is on the data that are collected, but specific types of citizen science can appeal more widely.

Both citizen science and work by professionals (i.e. forest managers and forest health professionals who are employed or contracted by relevant organisations concerned with forestry) can support monitoring and research on forest health (Fig. 1). Therefore, when considering forest pests and diseases, it is important to evaluate how and when citizen science can support, complement or augment the existing work undertaken by professionals.

The aim of this paper is to show the development and vision of citizen science in pest monitoring in forests. In the last decade, there has been an increased interest in citizen science and its potential in forest pest monitoring, especially in early detection of invasive pests. We have taken an integrated view of forest pest monitoring, where we not only focused on early detection, but we also took into account less investigated purposes such as early warning or monitoring of emerging and native pests, or their impacts. We will show the limitations and opportunities for citizen science and prepare recommendations for future projects for monitoring.

## Part 1: Opportunities for Citizen Science: Reflecting on Past Projects

**Fig. 2:**
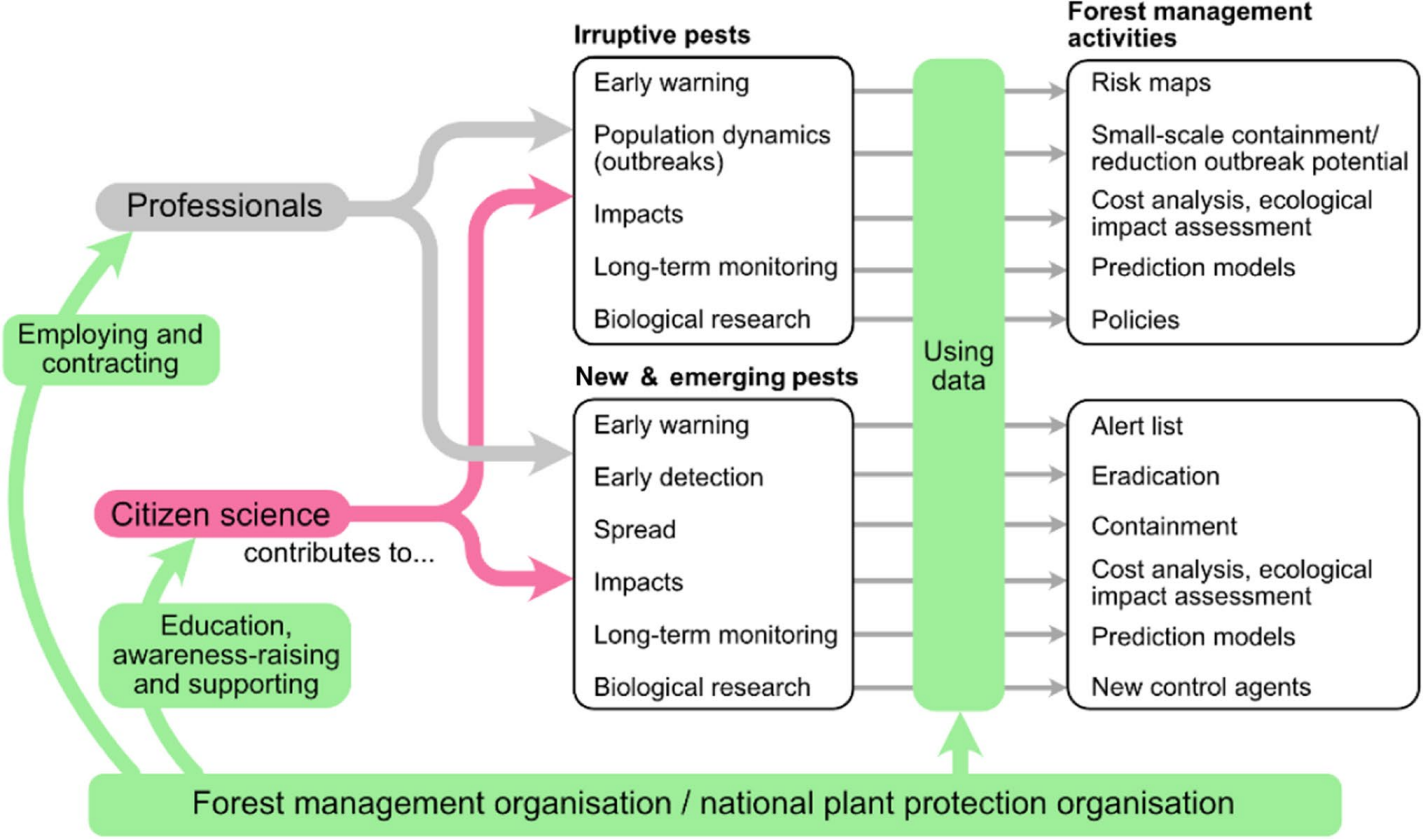
Professionals collect data to meet a wide range of needs regarding irruptive, new and emerging pests, but citizen science can complement and augment these data with support from relevant organisations, who can use all the available data for monitoring and to support forest management activities

Citizen science can be used in different ways to monitor forest pests, both irruptive pests and emerging and new pests, and has implications for forest management ([18]; Fig. 2, Table 1). For both irruptive pests and emerging and new pests, citizen science can be used for early warning, impacts, long-term monitoring and science (Fig. 2). However, in these cases, the motivation of the participants, the tools used and the forest management activities vary. For example, in early warning of outbreaks of irruptive pests, citizen science data can be used to prepare risk maps, while for emerging and new pests, citizen science information on pests outbreaks from other countries on native trees with similar climatic situations can be used for the preparation of alert lists of potential new or emerging pests. Monitoring population dynamics is a citizen science activity typical for irruptive pests, while early detection and monitoring of spread are more pertinent for emerging and new pests. Below, we discuss how citizen science can be embedded in three activities: early detection and early warning of new pests, early detection of outbreaks and impacts and biological research.

**Table 1:** Opportunities, limitations and recommendations on the use of citizen science for different purposes in forest pest monitoring and research

Goal	Opportunities	Limitations	Recommendations	Examples
Early warning	Use of sentinel trees Use of existing opportunistic citizen science databases Preparation of risk assessments and risk maps Prioritisation of new pest surveillance	Not all species are included Species may not be known to citizen scientists Existing databases can only be used for areas where there are a lot of potential participants	Increase number of observations in less well-recorded areas Greater use of data from opportunistic citizen science databases	[24, 25]
Early detection of new pests	Many people are in forests and can support early detection Citizen science activity tends to be greatest in areas with most introductions (e.g. urban or natural areas with high recreation)	Data are spatially biased Knowledge of species may be a limitation Some introduced species are relatively unknown and therefore professionals and scientists cannot provide information immediately Data verification costs may be high	Develop tools, e.g. smartphone apps, for submitting observations Develop awareness campaigns; use social media including passive surveillance Use of DNA sequencing methods	[26, 27•]
Early detection of outbreaks	Use of different new technologies Many people in the forest and can support early detection	Often unstructured data, biased (i.e. unevenly distributed) over space and time Keeping citizen scientists engaged over the long term Knowledge of certain species may be limited Sensitivity (poor detection probability) Specificity (over reporting which inundates regulatory bodies with false reports to follow up	Set up a project with structured sampling design Promote the use of new technologies, such as drones Use of simple methods and targeted detection tools, e.g. lures	[28, 29•]
Impact	Use of different new technologies Many people in the forest who could be involved in recording Large networks	Bias in assessment of damage (e.g. greater damage more likely to be reported) Often unstructured data, bias over space and time Keeping volunteers engaged Mostly damage is very non-specific and knowledge of species biology is necessary	Set up a project with structured design, possibly including site revisits Promote the use of new technologies Use of simple methods for assessing impact Use of bioblitzes	[30–32]
Research	Involvement of schools Sentinel trees Answering biologically relevant questions of forest pests Assessing species interactions Climate change research	Data quality needs to be sufficient More detailed studies can be undertaken	Develop protocols that are scientifically reliable for the analysis May require revisiting sites Statistical correction Education of the volunteers	[33, 34••, 35••]

### Early Warning and Early Detection of Invasive Pest Species

Box 1 Different types of citizen science projects that can be used for insect pest monitoring.

**Table:** 

Opportunistic (or passive, or unstructured) citizen science: Opportunistic citizen science projects are those in which biodiversity data are collected from volunteers when and where they choose, without data being captured according to specific recording protocols. Data on forest pests can be extracted from across the world, but because the records are usually presence-only based, they must be interpreted with caution. There are several platforms available to submit these records such as iNaturalist (www.inaturalist.org) and Observation.org (world.observation.org). The data can be used for surveillance of invasive and emerging pests Structured citizen science: Volunteers follow scientifically designed protocols at set times and/or places. By controlling the data collection process, the data are more useful for systematic monitoring, but this requires a higher level of commitment from volunteers, advance planning by scientists and long-term commitment from funders. These projects can be used for monitoring and hypothesis-driven research
Targeted species recording: Projects that aim to survey or monitor a particular species or species group in a certain area. For instance, LIFE ARTEMIS (www.invazivke.si; [36••]) is a citizen science project focused on the early detection of alien pests in the forests of Slovenia. In this project, a mobile app and a website were developed for recording the alien species and data were confirmed by experts. Whenever notable records were received, this was quickly communicated to the national authorities Bioblitz: A short period of surveying living organisms in a designated area [37]. The bioblitz gives a snapshot overview of the biodiversity in a short time, which can inform on-the-ground management and decision-making organisations on the presence of species and in the case of forest pest monitoring, new and emerging pests

Early warning and early detection of new and emerging pests are required to identify potential pests as soon as they move out of their natural range and to identify pathways of introduction and high-risk areas [38, 39••]. When an invasive pest species is detected for the first time, it has to be managed (and potentially eradicated) as soon as possible to avoid spread and potential impact on the ecosystem, economy and human health, and to assess their potential risks and ascertain potential management measures. Many plant health professionals are working in the field of early detection or risk management, and there are many examples of the success of early detection and consequent rapid response. One of the main highlights is the Asian longhorn beetle (*Anoplophora glabripennis*), which according to EPPO [40] has been found in many European countries but so far has always been successfully eradicated in Europe. However, there are also some examples where the species was not detected (or managed) early enough for successful eradication, like for Citrus longhorn beetle (*Anoplophora chinensis*) and Japanese beetle (*Popillia japonica*) in Italy [41, 42], the emerald ash borer (*Agrilus planipennis*) in Russia [43] or Oak Processionary moth (*Thaumetopoea processionea*) in the UK [44]. Citizen scientists can support early warning or early detection of species in forest and non-forest areas where there are trees (e.g. parks and gardens).

Early warning is the advance notice of species that could become a risk. Although early warning is an essential element of monitoring plant health using citizen science, this aspect is often under-promoted in scientific publications which deal with the surveillance of forest pests. In fact, volunteers who identify potentially invasive species can form an important early warning network for tree health [45, 46]. By regularly screening (social) media, scientific publications and citizen science databases like iNaturalist, new, emerging or recurring pests can be identified [25, 47••]. This information helps risk assessors and risk managers to prepare for and prevent possible outbreaks of new plant pests and diseases. By using this horizon scanning, the European Food Safety Authority (EFSA) and the European and Mediterranean Plant Protection Organisation (EPPO) identify pests and pathogens that might be of concern to the European territory [48, 49]. Citizen science can come into play here via passive observations, because plant health authorities often search large citizen science platforms for potential new pest species. On the basis of these data, plant health organisations like EPPO, for example, prepare an Alert List to draw the attention of its member countries to certain pests that may pose a risk to them and to provide early warning [24]. Species highlighted during this process should be assessed, and pathways of introduction should be identified. From the pathways and biology of the species, risk maps can be developed. Although there are many potentials for citizen science in early warning, there are not (yet) many projects which are practising early warning or horizon scanning (e.g. the monitoring and management of *Cerambyx cerdo* in the Mediterranean region [50]), so this provides a good opportunity for citizen science to grow in this area.

On the basis of the early warning, early detection can be organised more efficiently and be more focused. Unlike in early warning, citizen science has already been widely applied in the early detection of invasive alien pests and diseases [26, 27•, 32, 36••, 51–54, 55••, 56, 57] and is suggested as a low-cost method of surveillance [26, 55••]. For instance, Kline et al. [53] report that a citizen science initiative was set up in Curry Country (Oregon) for the early warning and detection of sudden oak death caused by *Phytophthora ramorum*. Similarly, Barker et al. [54] state that another citizen science initiative called the Forest Health Ambassador Program was established in Oakville (Ontario, Canada) for the early detection of invasive pests from the urban forest. Seidel et al. [58] state that drawing records of specific forest pest species of interest from existing citizen science databases and platforms would help to provide a better overview of the distributions of these taxa [58]. The awareness raised by citizen science tools, such as taxonomic interest forums, recording platforms (e.g. iNaturalist) and social media platforms, may, in the future, even help to act as an early warning system for these pests [58].

It is important to react as soon as possible after successful early detection of forest pest species (whether native or alien); otherwise, an outbreak can become uncontrollable. However, there are many people who visit forests and can report the start of an outbreak. For instance, Brown et al. [47••] reported that in the Observatree project, a network of tree health volunteers was created. They were trained to recognise the symptoms of attacked trees and to report them and were well supported with feedback to maintain their motivation and participation. Arboreta are often visited by the general public, and so there are many possibilities for citizen science related to early warning in these places, such as the International Plant Sentinel Network [59], which is using a network of arboreta and botanical gardens to identify pests on non-native tree species. Another opportunity is the use of novel methods, like eDNA, remote sensing and the introduction of sentinel trees, i.e. trees that are specifically monitored for their health and the presence of pests and diseases [36••, 60]. These methods can be used by citizen science under guidance of forest health experts, and therefore, a larger area can be covered, and more samples can be taken for the detection of invasive pests. The use of citizen science in the early warning and early detection of invasive pests offers many opportunities for citizen science to work in collaboration with professional plant health specialists.

In the past, not all invasive pests were found in time or were not prioritised, which negatively affected the eradication outcome [55••, 61]. To avoid this in the future, the availability of a centralised system where observations are collected by a competent authority is important so that rapid response actions can be taken immediately [36••, 39••]. To encourage early detection of invasive forest pests, awareness-raising campaigns and the development of submission apps should be stepped up. More attention also needs to be paid to areas from which few observations are made.

### Early Detection of Outbreaks (of Invasive or Native Species) and the Impacts of Pests

Monitoring pests and their impact is an important part of overall forest monitoring, and in many countries, it is undertaken by the forest management organisations. As with invasive species, it is important to detect outbreaks of native pests as soon as possible so that the requirement for action can be assessed. This means that there is value in many people on the ground surveying for potential outbreaks, which provides a clear role for citizen science. There are several citizen science projects in which people are surveying populations of irruptive and emerging pest species [28, 29•, 34••, 35••, 62]. For instance, Valdés-Correcher et al. [35••] focused on defoliator damage on oaks in order to investigate the interactions with other organisms. Similarly, Meentemeyer et al. [28] investigated the potential of citizen science to predict outbreaks of sudden oak death.

Surprisingly, less has been done on monitoring of abundance over time through citizen science. For this, relative or standardised measures of abundance or impact are valuable. Carleton and colleagues [29•] provided citizen scientists with pheromone traps to monitor the spruce budworm, which generated valuable spatio-temporal data for this species, and from which management implications could be inferred. Pocock and Evans [33] asked members of the public to record the degree of damage to horse chestnut (*Aesculus hippocastanum*) caused by the leaf-mining moth *Cameraria ohridella*. Another project focusing on schools and ecological networks in oak trees from all over Europe asked school children to send back leaves on which the infestation rate of defoliators (leaf chewers, miners and gallers) was assessed [35••]. If continued, this would be a good and simple method to monitor defoliators. In the Netherlands, the project ‘Natuurkalender’ is collecting data on the phenology of different groups of pest organisms. One of them is the emerging pest of the oak processionary moth (*Thaumetopoea processionea*), which has shown a strong spread in the recent years in the northwestern part of Europe.

Citizen science is often thought of as being most beneficial for getting lots of data from wide spatial extents via unstructured surveys (i.e. in which people collect data when and where they choose). Most previous projects only requested that citizen scientists record presence data on invasive alien species [27•, 36••, 63]. As a result, it is hard to distinguish places where the species is absent as there are locations where there are no observers. Although this could be statistically addressed in some cases, as de Groot et al. did for Oak lace bug (*Corythuca arcuata*) [64], an alternative approach is to standardise the monitoring. For example, ‘Observatree’ started a project on sentinel trees, in which volunteers adopt a tree and monitor it for potential invasive alien species and therefore also take into account the absence points [36••]. However, it can also be effective in providing data regularly over time, e.g. when the same sites are systematically surveyed over time (e.g. [65]). There is good potential for citizen science to deliver this type of data, which could provide high-quality data supporting the monitoring of pest outbreaks.

Monitoring the impacts of the pest is mostly focused on the health of the trees, and pests can be classified by their impact. Pests that cause low impact are those where no damage is observed and no intervention or management is required; medium-impact pest species cause damage and should be managed and controlled, but only in the short term, while high-impact species require management and control because they may have significant economic and environment effects [56]. Hence, in order to know the threat faced by forests, it is important to evaluate the state of the trees, and this is a valuable role for citizen science studies [28, 30, 32, 66••, 67]. One common practice is to register the symptoms of pest damage [30, 66••], or to develop a rigorous scoring system or index to record the health of the trees, e.g. stem and crown condition, allowing tree health evaluation to be comparable across space and time [52, 68, 69].

In addition, it is important to evaluate the impact on the biodiversity of the forest, but this is rarely considered in citizen science projects. Two citizen science studies [31, 70] demonstrated that the emerald ash borer, *Agrilus planipennis*, altered the community of insectivorous birds in the USA. Similarly, Valdés-Correcher et al. [35••] found that the herbivorous leaf gallers and leaf miners in oak trees (*Quercus robur*) were influenced by climatic factors and by leaf traits. These show the benefit of considering different aspects of the ecosystem, not just the insect forest pest or the tree health. They also demonstrate the value of sampling more variables at the same location to effectively understand the impacts of forest pests. Citizen science can also be used to identify host resistance to forest pests [71].

There are more opportunities in terms of tools and methods that may be useful for forest pest monitoring. In recent years, for example, drones have become very popular as a hobby, and this tool could also be used by citizen scientists to monitor, for instance, the pine processionary moth [72]. On the other hand, we can also learn a lot from other fields of study. Biodiversity conservation relies heavily on citizen science, and this involves monitoring the trends of butterflies, birds and other taxonomic groups in several countries [73, 74]. Methods such as transects, light traps or malaise traps applied in other projects [74–77] can also be used to monitor plant health to gain insight into the population dynamics of certain pest species. Organising dispersed bioblitzes (Box 1) is another innovative way of obtaining targeted biodiversity data in a certain area [37, 78]. It is also valuable to remember that citizen science projects can be used to record attributes of species, such as relative abundance or indices of impact, rather than simply presence [79].

### Research of Species Biology with CS

Many aspects of the biology of pest species and their impacts require professional engagement. However, when working on a large-scale, systematic and or lab-based projects, there are many opportunities for citizen science to contribute knowledge about these species and their interactions. Studying the biology of forest pests by citizen scientists is rather uncommon. However, such participatory, hypothesis-driven research allows researchers to address questions about the pest biology of a species at larger spatial scales and with greater temporal resolution than would otherwise be feasible [33, 80]. A robust example is the ‘Conker Tree Science’ project where the parasitism of the horse-chestnut leaf miner, *C. ohridella*, by naturally occurring parasitoid wasps was successfully studied by citizen science participants [33]. They found that both leaf damage of horse chestnut and parasitoid attack on leaf-mining moths were greatest where the leaf-mining month had been present the longest. Also, plant-herbivore-natural enemy interactions were studied by both citizen scientists and schoolchildren. In this way, the latitudinal and climatic effects on the relationships between insect herbivory, leaf chemistry and bird attack rates were studied across the European geographic range of the pedunculate oak (*Q. robur*) [35••]. They found that climatic factors explained the variation of gall makers and leaf miners, and not the variation of leaf damage, leaf defences and bird attack rates, and that leaf traits influenced both gall makers and leaf miners, whereas both leaf traits and bird attack rates did not vary with leaf damage. These results improve the understanding of the mechanisms driving geographical variation in the relationships between plant, herbivore and predators. In the Netherlands, the phenology project ‘de Natuurkalender’ (www.naturetoday.com) gives the phenology of the oak processionary moth over several years and provides the opportunity to investigate the influence of climate change on this species and its interactions with the phenology of its host.

An interesting opportunity is the use of sentinel trees by citizen science. Besides early warning of pests and diseases, other research questions can be investigated, such as increasing the understanding of known pests, identifying new pest-host associations, identifying potential biocontrol agents and supporting integrated management [59]. Another important underused part of citizen science is the study of species interactions [35••, 81]. These few examples show that studying the pest biology of forest health and their relationships with other species can be either the main or a secondary goal of citizen science projects.

Citizen science can also be a cost-effective way of gathering specimens from across a wide geographic range, with further biological analysis undertaken by professionals. In this way, new hosts for *Phytophthera ramorum* were identified from samples obtained during the Sudden Oak Death Blitz in California [82].

### Engaging People with Forest Health and Management

Informing and raising public awareness about forest health and forest pest insects is very important to promote sustainable forest management and use. In many cases, citizen science is used for data collection, but public engagement about forest health is a really valuable impact for citizen science on forest pests because of the role the public can play in surveillance, monitoring and research, but also because their actions can directly affect these drivers of change, e.g. the introduction and spread of invasive species.

Recently, several surveys have revealed that public knowledge of tree pests and diseases and the measures to control them are generally low, although it varies among stakeholder groups [83–86]. These surveys also point out that the level of concern and acceptance of management actions tends to be high when individuals are aware of the problem [83, 84], although there are preferences for different types of action. For instance, mechanical removal was preferred in a survey conducted in Slovenian forests [86] and biological control in a UK survey [84], and there is an overall lack of support for chemical control [84, 86, 87]. Support for management is high when species are invasive [86, 88], but this may not be the case when the pest or disease is native [89]. Increasing public knowledge about the impact and biology of native and invasive pests is essential in order to gain public acceptance of the most appropriate management measures for each pest.

Given the high importance of public dialogue and understanding about forest health, several citizen projects have emphasised volunteer education and provided educational materials and training to support ongoing learning [53, 90, 91]. Participating in a citizen science project increases people’s awareness, understanding and engagement around environmental issues such as forest health [30, 91–93]. Volunteer training also has a ‘multiplier effect’, as concerned participants in citizen science can become ‘science communicators’ and a ‘supplementary source of communication to the public’ [55••]. For example, in a volunteer programme for early detection of new invasive species by private landowners in Minnesota, the volunteers who took part in a learning module were more likely to recruit volunteers than those who only received an invitation letter [46].

Forest health professionals can also respond to public concerns, for example in Western Australia, the community was concerned about a forest disease, and the impetus to create a citizen project (‘The Marri Canker project’) came from the local landowners observing the decline in local Marri populations [32]. Public dialogue and cocreation of projects are important in these cases of existing public concern to create effective citizen science [72].

### Limitations in Citizen Science

In the previous sections, we have showed that there are many opportunities for citizen science in forest health. However, there are also limitations with citizen science, particularly when used as a tool for monitoring and research. The important limitations we have identified for citizen science data are limitations with species identification, concerns about data quality, uneven coverage of records in space and time and overall lack of fit-for-purpose of the dataset for its intended use.

The ability to correctly identify a species is a basic requirement for data on forest pests. Sometimes, actions need to be taken for particular species like the EU Quarantine species or alien pests that occur near the border of a country [36••], so accurate identification guides (e.g. [94]) to support correct identifications are important. Potential invasive or emerging pests may not be well known or sometimes not even known by science [95]. In the case of difficult taxa, it may be only very experienced people (professionals or expert amateurs) who can identify the species; sometimes, that may only be possible with molecular methods (and this may be especially so for disease-causing organisms, rather than pest invertebrates). There is also a bias in the traits that make species reportable or noticeable, with larger, more brightly coloured and strikingly patterned species being most likely to be reported [96]. To highlight pest species of concern, awareness-raising campaigns and identification guides are needed [36••]. Where possible, citizen science records can have confirmatory evidence (typically photographs, but could include sound recordings or specimens where appropriate) to allow species identification to be confirmed. Verifying records does require an investment in resources by the project organisers, although this task could also be carried out by trained volunteers [36••].

Species identification is only one component of data quality. Some critics express concern that citizen science data are of low quality [97], but there is a range of ways of ensuring data from citizen science are of good quality [98–100]. Different types of citizen science data can be collected, e.g. presence, abundance or attributes such as size [79]. These may vary in their precision and accuracy and, of course, also depend upon the skills and experience of the focal audience. An important aim is to ensure the quality of data acquired [33] and, if necessary, find ways to improve training, the quality of resources, or alter the methodology or even the aims of the study to ensure that quality is sufficient for the project’s purpose. For example, Castagneyrol et al. [34••] found that schoolchildren over-estimated the attack rate of artificial caterpillars through the citizen science project ‘Tree Bodyguards’. It was suggested that face-to-face courses would increase the quality of the data, or that scientists could reassess the data of the schoolchildren. Alternatively, Pocock et al. [33] considered data quality in a project on horse-chestnut leaf miner (*C. ohridella*). They used a simple, five-category method to facilitate accurate scoring of leaf damage, and from validated subsets of the data, they were able to statistically account for over-estimates of parasitoid numbers. The key requirement for citizen science is that the data are sufficiently accurate to be useful in the original project, although it is worth noting that many citizen science data can be reused in projects beyond the original one.

The limitation of uneven coverage of data is one that is often described, but in many cases, it is inevitable. Sometimes, this can be overcome with a highly structured design, but the restrictions of such projects typically lead to lower participation by volunteers. Citizen science then often focuses on the collection of unstructured data [26, 27•, 36••, 51–54, 55••, 56, 57] (Box 1), and these are typically biased towards areas with higher introduction risks (i.e. urban areas) [101–105]; thus, the biases can sometimes actually enhance the ability of citizen science to provide early detection. It is possible to statistically take account of these biases, although ideally, this should be planned at the inception of the study. Knowledge of these biases can support the provision of advice for where to search for pest species [64]. In theory, biases could arise intentionally driven by people’s concern about the use of the data if they deem forest management to be unacceptable [106••]. This should be highlighted through consultation at project inception and addressed through engagement and co-design.

Bias can also occur over time. Although citizen science is increasing, we should be concerned about the sustainability of people’s participation and also the risk of confusion in a plethora of projects. In order to improve the evaluation of the risk of alien forest pests to forest health, it is important to continuously monitor their impact on tree health over long periods of time. To this end, long-term projects [28] with an intensive monitoring network are required, as has already been proposed in early detection programmes [47••]. In California, for instance, the citizen science programme ‘Sudden Oak Death Blitz’ was carried out for 6 years [28]. To ensure that participants continue to participate over time, the evaluation of the impact on tree health may need to be simple, have complete and detailed guidelines [80], include intensive training and frequent interaction with the participants [54, 107, 108••], and may focus on the use of tools that facilitate data collection by participants, such as smartphone apps [66••, 107].

Thus far, we have considered the limitations of citizen science alone, but as part of an overall monitoring or research project, the relationship of citizen science to professional monitoring and research will need to be explored (Fig. 2). It is conceivable that forest health organisations could, in the future and for some specific purposes, rely almost entirely on citizen science (as is the case for national bird monitoring in countries like the UK). However, when considering the opportunties and limitations of citizen science (Table 1), professional monitoring will remain foundational for forest health organisations, but we expect that citizen science will have an increasingly valuable role in augmenting or complementing data from professionals to expand our current knowledge on forest pests.

Data flow can be an obstacle for some citizen science if the data are not shared efficiently. The data flow between citizen science projects and the professional organisation depends partly on whether or not the professional organisation organises the project. For instance, Observatree and LIFE ARTEMIS have developed projects for the early detection of forest pests and invasive alien species in the UK and Slovenia, respectively, with direct data flows to the national plant protection organisations [36••]. It is more difficult when the citizen science project is developed independently from the professional organisations like the citizen science platform iNaturalist, but Adriaens et al. [109] developed a pipeline to ensure data flow from GBIF on invasive alien species. However, this will be more of a limitation when citizen science platforms or projects are not freely accessible.

Overall, these concerns are about the fit-for-purpose of the citizen science data [20]. It is clear that the concerns of species identification, data quality and uneven coverage can (often) be addressed, but this should be done early in a project lifecycle and ideally through co-design or a testing phase prior to the project launch. This is part of the way of doing good citizen science as we go on to discuss in the following section.

## Part 2: Where Do We Go from Here?

### Ways of Doing Good Citizen Science

Before undertaking citizen science, it is important to critically consider the role and use of citizen science: citizen science will not always be the ideal approach to achieve the aims of a project organiser, whether those aims are for monitoring, for engagement or for both [85]. When citizen science is used, it needs to be designed to address the questions of interest [20, 110]. A formal cost–benefit evaluation of citizen science versus alternative approaches could be considered before developing or supporting citizen science [111]. However, it should be remembered that there are a myriad of citizen science approaches [75], so simply because one approach does not meet the requirements, other approaches may be more suitable [112]. Additionally, many citizen science projects have already been developed, so be aware that data from existing projects can be reused for additional purposes, thereby avoiding the excessive profileration of many different projects, which can confuse the citizen scientists. Communication and collaboration between project organisers should also help to reduce confusion for potential participants.

When considering doing good citizen science, it is important to consider the diversity of citizen science approaches (Box 1) and their pros and cons. Broadly speaking, contributory citizen science can range from unstructured to systematic, structured approaches [113], but with much variation in between. Unstructured citizen science, in which people take part when and where they choose, leads to uncontrolled spatial variation that needs to be accounted for statistically [102]. Projects should be designed with the analysis in mind to avoid data collection where biases cannot feasibly be accounted for. However, for these, there is the mass participation potential to have many ‘eyes on the ground’ which is ideal for early detection. In contrast, structured monitoring supports standardised data collection which is much closer to typical scientifically designed monitoring, but this requires a high degree of commitment from organisers and volunteers, and long-term commitment from funders (e.g. [74]). Semistructured approaches provide a valuable combination whereby participants take part when and where they choose, but do so in a relatively standardised way, e.g. following a simple protocol [114]. The Backyard Bark Beetles project in the USA (www.backyardbeetles.org) and Bearded Beetle Biodiversity in South Africa (https://citsci.co.za/beetles/) asked people to sample ambrosia beetles using standardised traps made from 2 L drinks bottles. One exciting opportunity is the way in which citizen science can be combined with professional monitoring to create an overall monitoring programme. This means that we can gain the benefits from citizen science (e.g. data collected at greater extents) while reducing the risk of relying solely on citizen science for monitoring and surveillance. This risk arises because volunteers are not obligated to take part in any project, and so could be affected by major societal issues such as the COVID-19 pandemic [115]. A blend of professional and citizen science monitoring can be designed to assess and control for spatial biases in citizen science, e.g. citizen science is more likely to take place in areas of high population density, near roads or in nature reserves [102], and so professionals can fill remaining gaps in a structured way. Additionally, new statistical approaches can be used to combine datasets, for example from structured monitoring (by citizens or by professionals) with opportunistic data [116].

Overall, it is good to adhere to the principles of good citizen science when designing citizen science, e.g. the 10 principles developed by the European Citizen Science Association [117], and to follow existing guides for good practice [118]. Understanding the motivation of participants is an important consideration when developing citizen science. People have a wide range of motivations for participating [119], and this will vary according to the subject of the citizen science project, e.g. long-term monitoring of insect population dynamics versus response to a newly arrived forest pest. Citizen science typically involves people who are relatively well-educated and are involved as an intentional pastime [22]. Some people will be motivated by a desire to connect with nature and do something useful, but when considering tree health, people’s sense of threat could be a strong motivator. For some participants, the opportunity to learn and gain new skills is an important motivator so should be considered in citizen science projects [120, 121]. Linking citizen science to local needs and local action could engage a wider range of participants [116], for example, local woodland owners and forest managers, who see the immediate value in collecting and sharing data for their own management practices. Designing citizen science projects with participants involved (so-called co-created projects) should increase their likelihood of having impact; this probably already happens frequently but is poorly documented because scientific outputs tend to focus on the biological results.

Co-design is a valuable approach to tailoring projects to different stakeholders. For example, early detection of invasive species is often intended for the general public, while early detection of outbreaks may be targeted at forest owners or other people who are regularly in the forest. Matching the scientific needs of a project to a specific audience should support the recruitment and retention of volunteers [119, 122]. It also allows for co-design with specific audiences, which is more effective than seeking to impose activities designed solely by scientists, even if this means adjusting the aims and outcomes from a purely ‘scientific’ perspective. This does not mean that citizen science is a poorer version of professional science; citizen science should, of course, be designed to provide sufficient scientific rigour to be scientifically useful. Co-design will also help to shape the activities around the concerns and needs of participants (where relevant), their skills, and help the teams to consider the ethical dimensions of citizen science and tree health at an early stage in projects [106••, 123]. This is important given the sometimes traumatic consequences of control after early detection of forest pests (e.g. [121]).

One aspect of the scientific usefulness of a citizen science dataset is ensuring the accuracy of data points as well as the fit-for-purpose of the whole dataset. There can be a trade off between ease of participation (and hence completeness of the data) and data quality [124], but this can be ameliorated through careful project co-design. Submitted data such as photos can be verified by professional expert taxonomists [36••] or by expert volunteers. Quality assurance of data points can be supported through volunteer training, which can be delivered in person or virtually [125]. It can also be supported through the verification of records by experts, or it can be automated, e.g. using image recognition where photographs have been submitted [126]. Data verification provides an opportunity for feedback to volunteers. Feedback about records and about the overall project is important for retention and their motivation [60, 127–129]. In general, regular communication to participants is valuable to boost their engagement [128].

A well-designed dataflow of the citizen science project is important to make citizen science useful, especially where rapid flow of positive records is required [75]. Technology can support good data flow through tools for data collection, such as websites and apps [130], so that, where appropriate, records can be sent to a responsible authority [36••, 66••, 107, 131]. This enables citizen science to meet the needs of organisers [18]. New automated identification technologies can be used to support rapid data verification [132, 133] and could even be used to automatically harvest records from social media sites [134]. Ultimately, data should adhere to the FAIR principles of findability, accessibility, inter-operability and reusability [135].

### Novel Technologies, Approaches and How to Implement Citizen Science in Data-Poor Regions

In recent decades, novel technologies for monitoring pests and diseases have developed rapidly [51]. Many of these technologies are now being used by professionals for the detection and monitoring of native forest pest populations and new pests. On the other hand, many of these technologies, such as the use of drones, have been used by the general public for recreational purposes. In order to utilise these new technologies, citizen scientists can be engaged to assist professionals in monitoring forest pests. Technologies, like drones or citizen sensing (i.e. citizens deploying sensors in the environment), can be applied by the citizen scientists themselves [136, 137] (Table 2). Species-specific lures are available for a range of forest pests (e.g. [138]) and could be particularly valuable because they do not require additional taxonomic expertise for verification of the species, and ethical concerns, e.g. of by-catch of non-pest species in generalised lures, are lessened. When deployed according to a protocol, lures can also provide assessment of population size. Furthermore, citizen scientists can crowd-source the collection of physical samples for laboratory analysis, e.g. inoculation studies [82] or DNA analysis. Professionals can optimise sampling design, and in collaboration with citizen scientists (in terms of co-design), pest monitoring by citizen scientists can provide high-quality data. Examples of this, albeit not related to forest health, are the American Chestnut Foundation which collaborates with scientists and citizen scientists for the collection of leaf tissue for DNA extraction to perform a species‐wide genomic analysis of American chestnut [66••], and an international citizen science project called ‘Pieris Project’ investigated the global invasion history of the agricultural pest butterfly (*Pieris rapae*) by collecting samples for DNA analysis from 32 countries worldwide [139].

**Table 2:** A selection of novel and potentially useful approaches for citizen science in forest pest monitoring

Novel citizen science approaches	Description
Environmental DNA	Collecting samples of substrate by citizen scientists in order to use environmental DNA for the detection of pests
DNA analysis of mixed samples	New methods like next generation sequencing are available to quickly sequence large mixed samples. These samples can be collected by citizen scientists using a variety of trapping methods
Unmanned aerial vehicles (UAVs, also called drones)	UAVs used at individual sites (in accordance with local regulations) for automatically collecting aerial images of forests. This can include multispectral imaging using specialist cameras
Remote sampling and risk maps—targeted visits	Create risk maps via remote sensing and species distribution models. These maps can be presented to citizen scientists to allow them to target their monitoring. (e.g. [45])
Dispersed bioblitzes	An intensive survey for a certain period of time in order to sample all living organisms in a designated area by citizen scientists dispersed over the whole country
Sentinel trees	Monitor native and non-native tree species for phenology and presence of pests and diseases in botanical gardens and arboreta all over the world as an early warning system for native areas of the tree species
Augment with professional monitoring	Combining citizen science data with the professional surveys, the total survey area will increase significantly with respect to forest health
Standardised citizen science design with repeated visits	Standardise sampling design for the monitoring of forest pest monitoring. Citizen scientists can take a certain area and repeatedly sample the pest insects throughout the year
Use of artificial intelligence (AI) and image classification	Automated image recognition with help of AI and image classification increases the potential to recognise new pest insect species and damage on trees. Having this tool would make it easier for the citizen scientist to identify, or guide the identification of, the species, and therefore raises awareness and educates the citizen scientist about the identified species
Citizen sensing	Use low-cost sensors to evidence forest pest detection/identification/activity, overseen and monitored by citizens
Lures	Physical or chemical lures that may be generalised or specific. Where the lures are species-specific, they do not require additional taxonomic expertise for confirming the forest pest

In many areas of environmental science, there is global variation in data available across the world, including from citizen science [79], and great variation in scientific and administrative infrastructures to support research and monitoring. This is also true for research and monitoring of forest pests and diseases. In this review, we have discussed the potential for citizen science to support monitoring and research for forest pests and diseases, so this raises the question: could citizen science be used in places that are currently data poor? From the examples of locally based forest monitoring [140] and arguments about citizen science and vector-borne disease control [141], we conclude that the answer is ‘yes’, citizen science does have a potentially important role. Ashephet et al. [141] state that the benefits of citizen science in data-poor regions are to boost data collection, tap into local knowledge and build partnerships. Citizen science could be particularly impactful where trees and forests directly link to people’s livelihoods and for sustainable development [142, 143], such as in community forests. The Plantwise programme is an interesting approach in which professionals collect data on crop pests by providing an advisory service to people in low- and middle-income countries and thereby collect data on pest impact and spread [144]. New citizen science opens the opportunity for data to be collected in data-poor regions, as demonstrated through citizen science of new data on zoonotic disease vectors in Uganda via participants who were trained and supported by professionals [145]. Here, expertise in citizen science from elsewhere in the world can be brought in, but Ashephet et al. argue that this expertise (often from the Global North) cannot be exported to the Global South, but it must be translated to local situations [141], via co-design to ensure acceptability for local stakeholders [106••, 142]. Indeed, when done well, this can be an effective and sustainable form of monitoring, as demonstrated by the example of ‘community-based monitoring’ for the past 2 decades [146]. The opportunities and recommendations for citizen science as discussed in this review could provide inspiration for designing successful, locally relevant citizen science in data-poor regions. With sufficient investment, this could transform the global monitoring of forest pests.

## Conclusions

In a changing world, pest monitoring is becoming very important due to the increasing numbers of new and emerging pests in forests. To increase the early detection of outbreaks and new pests, there should be many eyes in the field. Today, surveys are mostly done by forest health experts, but to increase the potential for early detection, citizen scientists are a welcome addition to monitoring and surveillance of forest pest insects. In the fields of forest protection, the main opportunities for citizen scientists are early warning, early detection of new pests and describing the impact of outbreaks. Additionally, we show that citizen scientists can help with scientific research, which is in many cases underused. Although there is a great potential for citizen science in pest monitoring, every field has its own limitations, opportunities and recommendations that need to be addressed. Therefore, tailored citizen science projects are needed to facilitate successful engagement by citizen scientists. At the moment, citizen scientists are most active in countries that have a strong culture of both citizen science and forest pest monitoring by professional forest health experts. In the future, we see great potential for citizen science in data-poor countries, where the forest health sector is less developed. In this sense, citizen scientists can help to understand and detect outbreaks of new pests faster and avoid problems in the future.
